# A Comparison of Cigarette Smoking Effects on Intervertebral Disc Cell Metabolism in a Rat Tissue Model

**DOI:** 10.1007/s10439-025-03958-x

**Published:** 2026-01-20

**Authors:** Nathan Buchweitz, Avery Madden, Joshua Kelley, Hui Li, Zhaoxu Meng, Michael Kern, Danyelle M. Townsend, Hai Yao, Yongren Wu

**Affiliations:** 1 Department of Bioengineering, Clemson University, Clemson, SC USA; 2 Department of Mechanical Engineering, Clemson University, Clemson, SC USA; 3 Department of Regenerative Medicine & Cell Biology, Medical University of South Carolina, Charleston, SC USA; 4 Department of Drug Discovery and Biomedical Sciences, Medical University of South Carolina, Charleston, SC USA; 5 Department of Orthopaedics and Physical Medicine, Medical University of South Carolina, Charleston, SC USA

**Keywords:** Intervertebral disc, Cigarette smoking, Energy metabolism, Finite element model

## Abstract

**Purpose:**

This study investigated the direct (cigarette smoke extract, CSE) and indirect (low nutrient) effects of cigarette smoking on intervertebral disc cell energy metabolism, with a focus on glucose consumption and lactate production in lumbar discs.

**Methods:**

Lumbar IVDs from Sprague–Dawley rats were harvested and dissected into nucleus pulposus (NP), annulus fibrosus (AF), and cartilaginous endplate (CEP) regions. Minced tissue cultures from each region were exposed to physiological control (5.5 mM glucose, 5% oxygen), CSE-treated (physiological + 10% CSE), or low-nutrient conditions (1.5 mM glucose, 1% oxygen). Glucose consumption rates (GCR) and lactate production rates (LPR) were measured using a biochemical analyzer. A finite element model was developed to simulate nutrient transport and adenosine triphosphate (ATP) synthesis in the IVD under experimental conditions.

**Results:**

Both CSE and low-nutrient conditions significantly reduced GCR and LPR in AF and NP, where NP cells exhibited the greatest metabolic activity. Low-nutrient conditions increased the LPR:GCR ratio, indicating an increase in glycolysis. CEP metabolism was marginally impacted by treatments. Computational modeling revealed that CSE conserved oxygen but reduced ATP synthesis, while low-nutrient conditions severely depleted glucose, oxygen, and ATP. Combined effects of CSE and nutrient deprivation exacerbated the reduction in ATP availability.

**Conclusions:**

Cigarette smoking impairs IVD cellular energy metabolism through both direct toxic exposure and indirect nutrient deprivation mechanisms, with the modeled low-nutrient conditions having a more pronounced effect. The NP is the most metabolically sensitive region in the IVD, while the CEP is more resilient to fluctuations with its environment. These findings provide insights into IVD metabolic adaptations to smoking.

**Supplementary Information:**

The online version contains supplementary material available at 10.1007/s10439-025-03958-x.

## Introduction

Cigarette smoking among U.S. adults has declined in prevalence from previous decades to approximately 11.5%, but remains the leading preventable cause of disease, disability, and death [1, 2]. Beyond its well-documented role in respiratory and cardiovascular diseases [3–6], smoking is implicated as a significant risk factor for musculoskeletal disorders, especially low back pain and intervertebral disc (IVD) degeneration [7–15]. Clinical studies involving magnetic resonance imaging have shown more severe IVD degeneration grades in individuals with a history of smoking or passive smoke exposure compared to non-smokers [12, 16, 17]. There is some evidence that structural disc abnormalities, such as annular tears and Modic changes, are also more prevalent among smokers [18, 19]. Moreover, for many smokers, increased pain sensitivity and recovery complications following spinal surgeries are noted [20].

Cigarette smoking is thought to contribute to IVD degeneration via two main mechanisms: an indirect pathway involving impaired nutrient supply to the IVD, and a direct pathway involving the exposure of disc cells to toxic chemicals in cigarette smoke [7]. As avascular tissues, IVDs rely on solute diffusion for the exchange of nutrients (e.g., oxygen and glucose) and metabolic wastes (e.g., lactate) with surrounding blood vessels [21, 22]. Smoking may disrupt this exchange by inducing vascular changes, including arterial vasoconstriction mediated by nicotine, as well as the recession of capillary networks near the IVD endplates and annular boundaries [7, 14, 23, 24]. Our recent in vivo studies using Sprague–Dawley (SD) rats have also demonstrated that smoke exposure can trigger aberrant calcification and remodeling of IVD cartilaginous endplates, affecting solute diffusivity within this region, and potentially further hindering nutrient transport to the disc [14, 25]. Additionally, cigarette smoke can reduce arterial oxygen availability by increasing carbon monoxide concentrations in the bloodstream, which binds to hemoglobin with greater affinity than oxygen [7, 26].

The direct pathway involves exposure of cells to toxic compounds in cigarette smoke, including nicotine, reactive oxygen species (ROS), and other oxidants, disrupting cellular homeostasis [3, 7]. In vitro studies have revealed that nicotine exposure alters IVD cell morphology and can reduce proliferation in a dose-dependent manner [7, 9, 27]. Moreover, nicotine exposure is found to suppress glycosaminoglycan and collagen synthesis, as well as promote fibrotic remodeling through increased Type I collagen synthesis [7, 27]. There is evidence that non-nicotine toxins found in cigarette smoke extracts (CSE), comprising thousands of chemical constituents, further reduce cell viability and metabolic activity more severely than nicotine alone [7, 9]. Additionally, smoke-induced oxidative stress is known to cause lipid peroxidation, protein oxidation, and DNA damage, compromising cell membrane integrity and viability [3, 28–30]. Through in vivo studies, smoke exposure has also been shown to elevate inflammatory mediators (e.g., IL-1β) and matrix-degrading enzymes, which drive catabolic imbalance within the disc [7, 31, 32].

Despite extensive research on the negative impacts of cigarette smoking on IVD tissues, the relative contributions of direct and indirect smoking effects on disc cellular energy metabolism are less understood. Rates of oxygen and glucose consumption, as well as lactate production, have variously been reported in IVD nucleus pulposus (NP) and annulus fibrosus (AF) cells [33–41]. The relationship between glucose and lactate metabolism, in particular, has been shown to be an important indicator of disc cell viability and matrix biosynthetic activity [34]. However, while the effects of varied nutrient environments on these parameters have been quantified, to the authors’ knowledge, no studies have compared these effects with the direct impacts of cigarette smoke exposure. Furthermore, there is a relative lack of characterization of cell metabolic rates in the cartilaginous endplate (CEP) of the IVD, which represents a critical nutritional pathway [37, 42]. This study aims to quantify the effects of cigarette smoking on IVD cell metabolism, isolating both (1) direct chemical exposure via CSE and (2) nutrient deprivation simulating impaired vascular supply. Using an in vitro tissue culture model of SD rat lumbar discs, region-specific glucose consumption and lactate production rates were determined under physiological and smoking-simulated conditions. We hypothesized that both mechanisms would suppress metabolic activity and exhibit glycolysis-dominant energy production, as indicated by increased lactate produced relative to glucose consumed. To complement the in vitro experiments, a finite element model was developed to simulate nutrient transport and adenosine triphosphate (ATP) synthesis under identical conditions. It was hypothesized that both effects would reduce ATP production. By integrating in vitro measurements of IVD nutrient metabolism with computational modeling, this study provides insights into how smoking-related stressors impair IVD cell energy metabolism, offering further context for smoking-associated IVD degenerative changes.

## Methods

### Disc Sample Preparation

Spines from eighty Sprague–Dawley rats (50 female, 30 male, 1.59 ± 0.68 months of age) were obtained as shared tissues from other IACUC-approved neurological research studies at the Medical University of South Carolina (see Acknowledgements). Animals were healthy, and their use in other studies did not have anticipated effects on the IVD or musculoskeletal system. Rats were housed in a 25 °C environment with a 12-h light/dark cycle and given ad libitum access to food and water. Euthanization was performed using an isoflurane inhalation method (PHR2874, Sigma-Aldrich, Darmstadt, Germany). Afterward, each animal was placed in the prone position and sprayed with 70% ethanol to minimize fur contamination. Lumbar vertebrae (L1–L6) were exposed via a posterior longitudinal incision, and spinal columns were extracted using a sterile scalpel and forceps.

**Fig. 1:**
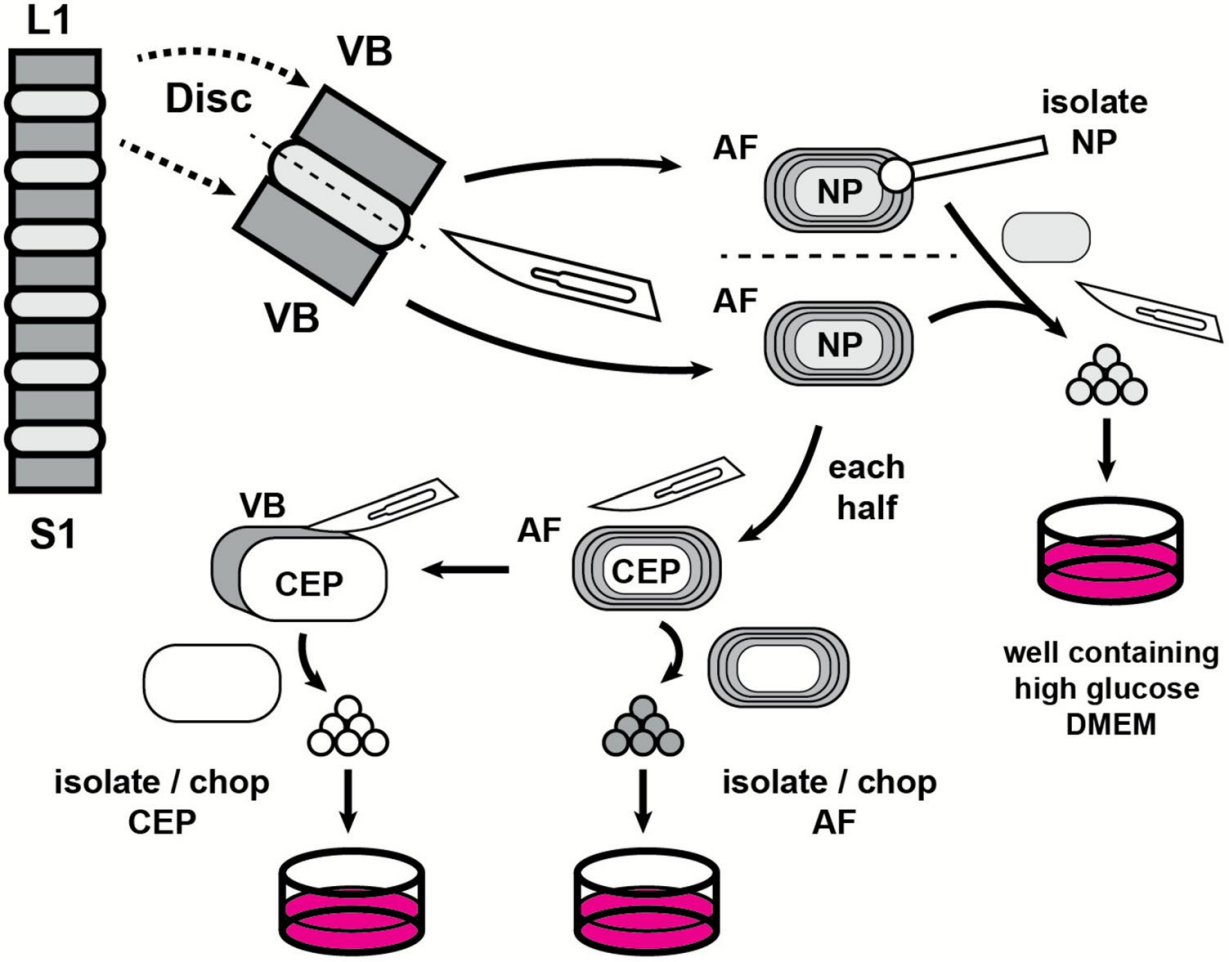
Schematic depicting the preparation of IVD samples from SD rat lumbar discs for in vitro metabolic rate measurement

Tissue specimens were immediately submerged in warm high-glucose (25 mM) Dulbecco’s Modified Eagle Medium (DMEM) and transferred to a sterile fume hood for dissection. Disc components (NP, AF, and CEP) were manually isolated based on structural and visual characteristics (Fig. 1). Discs were cut transversely through the mid-plane to expose the NP, which was collected with a blunt probe. The surrounding AF, identified by its lamellar structure, was separated from the NP and CEP using a No. 10 blade scalpel. The CEP was distinguished by its opaque, pearl-gray appearance and dissected carefully to avoid contamination with bone. Isolated tissues were then minced into ~ 0.5-mm pieces to minimize nutrient gradients and were homogenized by region across the lumbar disc levels [38]. Approximately equal weights (50–100 mg) of tissue were allocated to culture wells for duplicate or triplicate measurements of metabolic rates within each IVD region.

### Preparation of Cigarette Smoke Extract (CSE)

Cigarette smoke extract (CSE) was prepared fresh every three days following established protocols [43]. Standardized research cigarettes (3R4F; University of Kentucky) were burned using a custom syringe-based apparatus in accordance with ISO 3308. The smoke was bubbled through 22.5 mL of clear 5.5 mM glucose DMEM, and the resulting solution was filtered through a 0.20 µm sterile filter (Thermo Scientific) to remove particulates. Nitrite concentrations were standardized using a plate reader absorbance assay at 320 nm, where optical density values were recorded to be approximately 0.8 [43]. CSE stock was diluted to a 10% treatment solution using additional DMEM containing 5.5 mM glucose, achieving concentrations equivalent to smoking 27 cigarettes per day, consistent with prior in vitro tissue models and relevant to recent US Census data [43, 44].

### Metabolic Rate Quantification

Prior to the experiments, tissue samples were preconditioned for 12 h in wells containing high-glucose DMEM (25 mM) and physiologically relevant dissolved oxygen levels (5%, ${P}_{{{\text O}_{2}}}$ = 0.05 atm). Tissue cultures were then randomly assigned to three primary treatment groups: physiological control (5.5 mM glucose, 5% O_2_), CSE-treated (5.5 mM glucose + 10% CSE, 5% O_2_), or low-nutrient conditions (1.5 mM glucose, 1% O_2_). As a secondary focus of the study, decoupling the impacts of glucose and oxygen deprivation on IVD metabolism, two additional conditions were tested (1.5 mM glucose with 5% O_2_, and 5.5 mM glucose with 1% O_2_). All groups were cultured at 37 °C in a humidified incubator. Each well received 800 µL of media for AF and CEP samples, and 600 µL for NP samples, based on preliminary testing to ensure that critical glucose levels (< 0.5 mM) for cell viability were not recorded during the testing period.

To determine metabolic rates, glucose and lactate concentrations in culture media were measured hourly for five hours using a YSI 2700 Select Biochemistry Analyzer (YSI Inc., OH). Detection ranges were 0–25 g/L for glucose and 0–2.67 g/L for lactate from 10 µL samples. Baseline readings (*t* = 0 h) were taken immediately after preconditioning. Tissue volume (${V}_{\mathrm{tissue}}$) was calculated using Archimedes’ principle based on measurements of wet weight (${W}_{\mathrm{wet}}$) and buoyant weight when submerged in 10 × PBS (${W}_{\mathrm{PBS}}$; ${\rho }_{\mathrm{PBS}}$ = 1.07 g/cm3 and pH = 7.4), as shown in Eq. 1. Buoyant weight measurements were performed following the tissue culture experiments.

1 $${V}_{\mathrm{tissue}}=\frac{{W}_{\mathrm{wet}}-{W}_{\mathrm{PBS}}}{{\rho }_{\mathrm{PBS}}}$$

Changes in solute concentration ($\Delta c$) were multiplied by the media volume (${V}_{\mathrm{media}}$) and divided by the observation time interval ($\Delta t$=1h) to calculate metabolic rates. These values were then normalized to regional IVD cell number, estimated from tissue volume (${V}_{\mathrm{tissue}}$) and published histological measurements of cell density (${\rho }_{\mathrm{cell}}$) in Sprague–Dawley rats [14]. The calculations used to determine reported glucose consumption rates (GCR) and lactate production rates (LPR) are detailed in Eq. 2:

2 $$GCR\ or\ LPR= \frac{\Delta c*{V}_{\mathrm{media}}}{\Delta t*{{\rho }_{\mathrm{cell}}*V}_{\mathrm{tissue}}}$$

To capture steady-state metabolic activity, GCR and LPR were averaged across time points from 2 to 5 h. Replicate measurements for each region were finally averaged within each animal for reporting.

### Data Analysis and Statistics

The effects of smoking treatment, IVD region, and their interaction on metabolic rate outcomes (GCR, LPR, and their ratio LPR:GCR) were analyzed using R software [45]. As tissue samples from different IVD regions were matched by animals, a mixed-effects model was fit to the data, considering unique animals as a random effect. Post hoc comparisons were performed both within each IVD region among treatment groups and for each treatment group between IVD regions, with *p*-values adjusted using the Bonferroni method [46]. Treatment group means and 95% confidence intervals estimated for each disc region are reported in Table 1. To further evaluate the combined influence of glucose and oxygen level on metabolic rate outcomes, a separate two-way ANOVA was conducted within the subset of samples not exposed to CSE (summary data for the interaction between glucose and oxygen levels are available in Online Resource 1, Supp. Table 1). The minimum significance threshold was set at *p* < 0.05 for all statistical tests.

**Table 1:** Glucose consumption rates and lactate production rates compared for various animal and human IVD tissues (reported as mean [95%-CI], mean ± SD, or range of values as available)

Species & IVD region	Glucose (mM)	Oxygen (%)	GCR (nmol/million cells/h)	LPR (nmol/million cells/h)	Ratio (LPR:GCR)	References
SD rat-AF	5.5	5	80.57 [62.68, 98.46]	94.89 [69.33, 120.45]	1.25 [1.03, 1.47]	This study
SD rat-NP	5.5	5	149.13 [131.25, 167.02]	195.3 [169.74, 220.86]	1.3 [1.08, 1.52]	This study
SD rat-CEP	5.5	5	40.23 [23.9, 56.56]	54.65 [31.34, 77.95]	1.36 [1.16, 1.55]	This study
SD rat-AF	5.5 + 10%-CSE	5	49.16 [32.83, 65.49]	71.09 [47.79, 94.4]	1.52 [1.32, 1.71]	This study
SD rat-NP	5.5 + 10%-CSE	5	81.74 [65.41, 98.07]	114.47 [91.16, 137.77]	1.45 [1.26, 1.65]	This study
SD rat-CEP	5.5 + 10%-CSE	5	26.6 [9.59, 43.6]	40.03 [15.75, 64.32]	1.49 [1.28, 1.7]	This study
SD rat-AF	1.5	1	24.07 [7.5, 40.64]	38.51 [14.81, 62.22]	1.62 [1.42, 1.83]	This study
SD rat-NP	1.5	1	52.13 [37.44, 66.83]	92.97 [71.97, 113.96]	1.83 [1.65, 2.01]	This study
SD rat-CEP	1.5	1	18.27 [3.05, 33.49]	26.77 [5.01, 48.52]	1.55 [1.37, 1.74]	This study
Bovine NP	5	1–21	–	5.5–9.5*	–	[38]
Bovine AF	5	1–21	–	4.5–10*	–	[38]
Porcine NP (notochordal)	5	21	205 ± 16	355 ± 21	1.7	[36]
Bovine NP (mature)	5	21	95 ± 8	204 ± 9	2.1	[36]
Bovine NP	0.4–0.7	–	10–120	–	2.01 ± 0.07	[33]
Bovine NP	1.0–5.0	2.5–17.5	–	75–200	2.01 ± 0.07	[33]
Bovine NP	0–5	0–21	–	240–275	–	[34]
Porcine-NP	1	5	~ 50	–	–	[40]
Porcine-NP	5	5	~ 110	–	–	[40]
Porcine-NP	25	5	~ 50	–	–	[40]
Porcine-NP	1	20	~ 40	–	–	[40]
Porcine-NP	5	20	~ 50	–	–	[40]
Porcine-NP	25	20	~ 80	–	–	[40]
Porcine-NP (notochordal)	5	1	~ 250	–	–	[39]
Porcine-NP (notochordal)	5	5	~ 300	–	–	[39]
Porcine-NP (notochordal)	5	21	~ 250	–	–	[39]

*Units provided in µmol/g dry wt/h

### Finite Element Model of the Disc Nutrient Environment

A finite element model was developed to compare steady-state distributions of nutrients and ATP synthesis in the IVD, as predicted by variations in cell metabolic rate under direct and indirect smoking effects. The model focused exclusively on nutrient transport and metabolism, without mechanical deformation. Continuity for nutrient solute diffusion was modeled from Fick’s second law under steady-state conditions (Eq. 3):

3 $${-\nabla \cdot (D}_{i}\nabla {C}_{i})={Q}_{i}$$

where ${D}_{i}$, $\nabla {C}_{i}$, and ${Q}_{i}$ represent the diffusivity, solute concentration gradient, and consumption or production rate of solute species $i$, respectively.

Glucose consumption rates and lactate production rates were incorporated from experimental data using a Michaelis–Menten chemical kinetics model (Eqs. 4, 5). Curve-fitting was performed on GCR and LPR averages measured under 1.5 mM, 5.5 mM, and 25 mM glucose media conditions (see Table 2 for ${V}_{\mathrm{max}}$ and ${k}_{\mathrm{m}}$ parameters of the model). GCR and LPR averages under the 25 mM glucose condition are provided in Online Resource 1, Supp. Table 1, and representative curve-fits are shown in Supp. Fig. 1. This process was conducted separately for data obtained under 1% and 5% oxygen environments, then linearly interpolated in the model based on local predictions of oxygen concentration.

**Table 2:** Finite element modeling parameters

Region	Oxygen (%)	GCR			LPR			Cell density (million-cells/mL)	Nutrient diffusivity		
		*V*_max_ (nmol/million-cells/h)		*K*_m_ (mM)	*V*_max_ (nmol/million-cells/h)		K_m_ (mM)		*D*_glucose_ (m^2^/s)	*D*_lactate_ (m^2^/s)	*D*_oxygen_ (m^2^/s)
		No CSE	10% CSE		No CSE	10% CSE					
AF	1	77.37	45.07	3.61	94.50	66.01	1.94	53.57	4.39E−10	5.80E−10	1.16E−09
	5	131.72	76.72	2.65	137.64	96.14	1.04				
NP	1	95.69	53.37	1.37	118.19	69.97	0.37	35.71	6.47E−10	8.54E−10	1.71E−09
	5	285.63	159.31	4.12	257.04	152.17	1.43				
CEP	1	39.64	25.36	2.13	41.93	28.76	0.62	78.57	5.18E−10	6.83E−10	1.37E−09
	5	116.63	74.59	11.10	126.27	86.61	7.78				

Presently reported in vitro metabolic rate measurements (GCR and LPR) were curve-fit to the Michaelis–Menten equation (see Online Resource 1, Supp. Fig. 1 for representative fits). Nutrient diffusivities were estimated based on solute molecular weight and fluorescein diffusivity measurements previously obtained for SD rat IVDs (see Online Resource 1, Supp. Note 1 for calculation [25, 47–49]). Cell densities were estimated from prior histologic characterizations in SD rat IVDs

4 $${Q}_{\mathrm{glucose}}=-{\rho }_{cell}\cdot \frac{{V}_{\mathrm{max}}^{\mathrm{glucose}}\cdot {c}_{\mathrm{glucose}}}{{k}_{m}+{c}_{\mathrm{glucose}}}$$

5 $${Q}_{\mathrm{lactate}}={\rho }_{\mathrm{cell}}\cdot \frac{{V}_{\mathrm{max}}^{\mathrm{lactate}}\cdot {c}_{\mathrm{glucose}}}{{k}_{m}+{c}_{\mathrm{glucose}}}$$

Oxygen consumption and ATP synthesis rates were coupled with GCR and LPR using previously validated stoichiometric relationships derived from the tricarboxylic acid cycle and associated downstream redox reactions in cell mitochondria [50]. These relationships are given in Eqs. 6 and 7:

6 $${Q}_{\mathrm{oxygen}}=\left\{\begin{array}{c}3\cdot {Q}_{\mathrm{lactate}}+6\cdot {Q}_{\mathrm{glucose}}\\ 0; ({Q}_{\mathrm{lactate}}>-2\cdot {Q}_{\mathrm{glucose}})\end{array}\right.$$

7 $${Q}_{\mathrm{ATP}}=-2\cdot {Q}_{\mathrm{glucose}}-6\cdot {Q}_{\mathrm{oxygen}}$$

IVD geometry was idealized as a cylinder with a radius of 1.5 mm and a height of 1 mm. Within this geometry, the NP region was defined with a radius of 0.85 mm, and CEP regions were partitioned with a thickness of 0.2 mm. The domain was discretized into 22,655 nodes and 21,120 hexahedral volume elements. The model geometry and mesh are depicted in Online Resource 1, Supp. Fig. 2. The exposed surfaces of the model, including the peripheral boundary of the AF and both the superior and inferior CEP-vertebrae interfaces, were prescribed fixed solute concentrations to mimic the tissue culture media conditions (${c}_{0}$). Two distinct sets of boundary conditions were assigned to simulate physiological and low-nutrient treatment conditions. In the physiological case, ${c}_{0,\mathrm{glucose}}$ = 5.5 mM, ${c}_{0,\mathrm{oxygen}}$ = 0.054 mM (5%, calculated from an estimates of oxygen solubility at 37 °C, Henry’s Law Constant = 929.6 atm/M [51]), and ${c}_{0,\mathrm{lactate}}$ = 0.9 mM (based on an assumed media pH 7.4). In the low-nutrient case, ${c}_{0,\mathrm{glucose}}$ = 1.5 mM, ${c}_{0,\mathrm{oxygen}}$ = 0.011 mM (1%), and ${c}_{0,\mathrm{lactate}}$ = 0.9 mM. Effects of CSE were modeled in both environments by augmenting metabolic rates proportionally according to the average differences observed in GCR and LPR relative to physiological controls (Table 2). A summary of model parameters, including metabolic rate constants (Vₘₐₓ and Kₘ), nutrient diffusivities, and regional cell densities, is provided in Table 2. Simulations and post-processing operations were performed using COMSOL Multiphysics (v5.6, COMSOL Inc., Burlington, MA).

## Results

### Effects of Smoking Treatment on IVD Metabolism

**Fig. 2:**
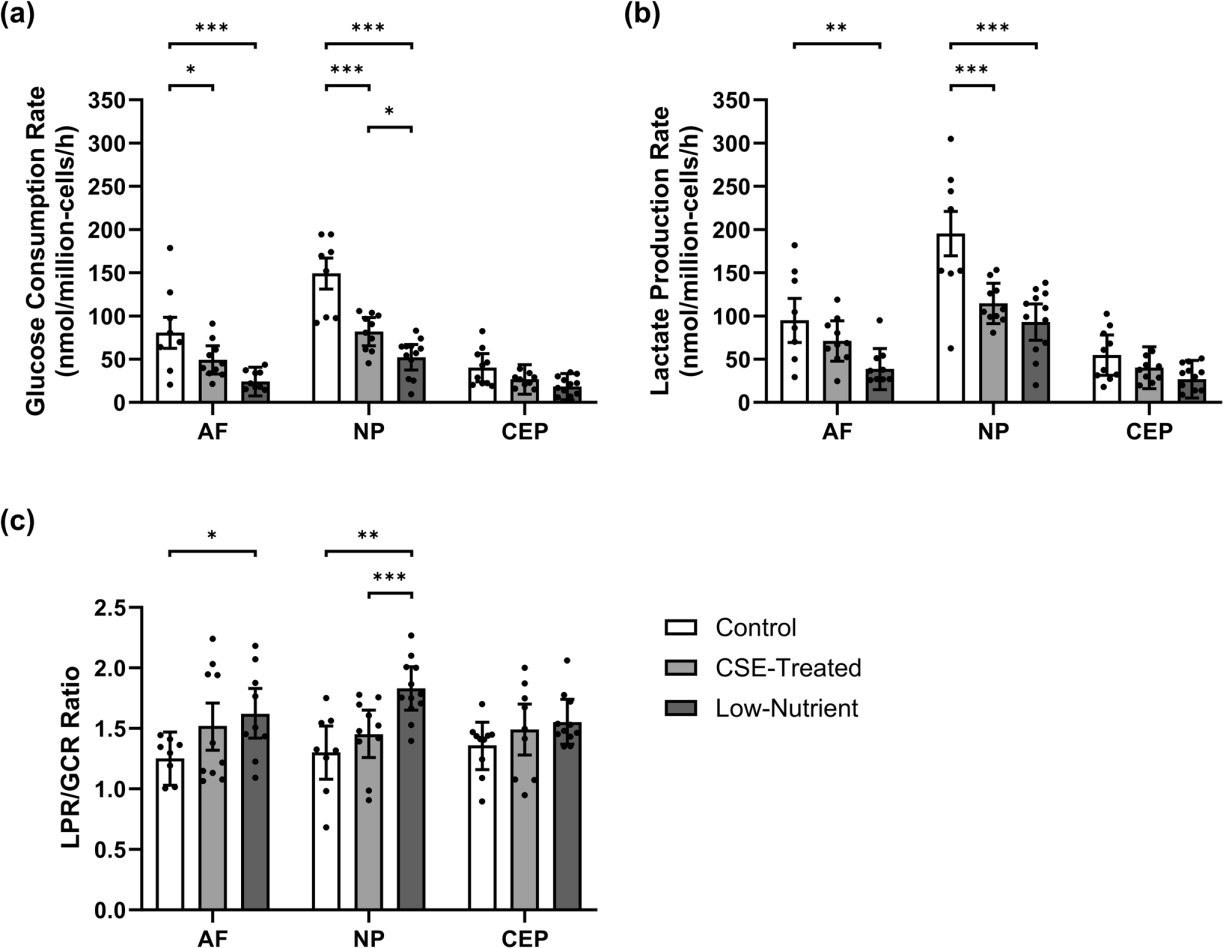
In vitro assessment of direct (CSE treatment) and indirect (low-nutrient) effects of smoking on IVD cellular energy metabolism in SD rat discs: **a** glucose consumption rate (GCR), **b** lactate production rate (LPR), and **c** cellular energy ratio (LPR:GCR). Each data point represents an average of 2- to 3-well plate replicates measured from a single rat. Asterisks indicate differences at the specified levels of statistical significance: *p < 0.05, **p < 0.01, ***p < 0.001

Both glucose consumption rate (GCR) and lactate production rate (LPR) demonstrated statistically significant interactions between the smoking treatment and IVD region (GCR: *p* < 0.0001; LPR: *p* = 0.0017). This suggests that the effects of smoking on metabolic activity vary by region. In the AF and NP disc regions, GCR was lower in both the CSE group and the low-nutrient group relative to the control (Fig. 2a). Comparing the CSE group with the control, these differences were as follows: Δ = − 31.4 nmol/million cells/h, *p* = 0.0355 in the AF, and Δ = − 67.4 nmol/million cells/h, *p* < 0.0001 in the NP (Fig. 2a). Comparing low-nutrient conditions with the control, these differences were as follows: Δ = − 56.5 nmol/million cells/h, *p* = 0.0001 in the AF, and Δ = − 97.0 nmol/million cells/h, *p* < 0.0001 in the NP (Fig. 2a). Furthermore, in the NP, GCR was significantly lower under low-nutrient conditions compared with CSE treatment: Δ = − 29.6 nmol/million cells/h, *p* = 0.0272 (Fig. 2a). Differences in GCR with treatment were not apparent in the CEP (Fig. 2a). In the NP, a similar response was observed for LPR, which was found to be lower under CSE treatment compared to the control: Δ = − 80.8 nmol/million cells/h, *p* < 0.0001 (Fig. 2b). Low-nutrient conditions also resulted in a lower LPR compared to control in both AF and NP regions: Δ = − 56.4 nmol/million cells/h, *p* = 0.0057 in the AF, and Δ = − 102.3 nmol/million cells/h, *p* = 0.0355 in the NP (Fig. 2b). Treatment effects were likewise not evident for LPR in the CEP.

Regionally, both GCR and LPR were higher in the NP than in the AF, regardless of treatment (Fig. 2a, b). For GCR, the differences were as follows: Δ = 68.6 nmol/million cells/h, *p* < 0.0001 in the control; Δ = 32.6 nmol/million cells/h, *p* = 0.0018 with CSE treatment; and Δ = 28.1 nmol/million cells/h, *p* = 0.0099 under low-nutrient conditions. For LPR: Δ = 100.4 nmol/million cells/h, *p* < 0.0001 in the control; Δ = 43.4 nmol/million cells/h, *p* = 0.0042 with CSE treatment; and Δ = 54.5 nmol/million cells/h, *p* = 0.0004 under low-nutrient conditions. GCR was also consistently higher in the NP than in the CEP: Δ = 108.9 nmol/million cells/h, *p* < 0.0001 in the control; Δ = 55.1 nmol/million cells/h, *p* < 0.0001 with CSE treatment; and Δ = 33.9 nmol/million cells/h, *p* = 0.0008 under low-nutrient conditions. Following a similar pattern, for LPR, these differences were as follows: Δ = 140.7 nmol/million cells/h, *p* < 0.0001 in the control; Δ = 74.4 nmol/million cells/h, *p* < 0.0001 with CSE treatment; and Δ = 66.2 nmol/million cells/h, p < 0.0001 under low-nutrient conditions. Uniquely, under control conditions, both GCR and LPR were modestly lower in the CEP compared to the AF: Δ = − 40.3 nmol/million cells/h, *p* = 0.0003 for GCR, and Δ = − 40.2 nmol/million cells/h, *p* = 0.0163 for LPR.

No treatment and region interactions, or regional effects alone, were apparent for the LPR:GCR ratio (*p* = 0.2405 and *p* = 0.6030, respectively); however, the treatment effect was significant (*p* = 0.0028). Compared to the control, higher ratios were observed in the low-nutrient group: Δ = 0.38, *p* = 0.0451 in the AF and Δ = 0.53, *p* = 0.0011 in the NP (Fig. 2c). In the NP, the low-nutrient group also exhibited a higher ratio compared to the CSE treatment group: Δ = 0.38, *p* = 0.0187 (Fig. 2c).

### Effects of Glucose and Oxygen Environment on IVD Metabolism

**Fig. 3:**
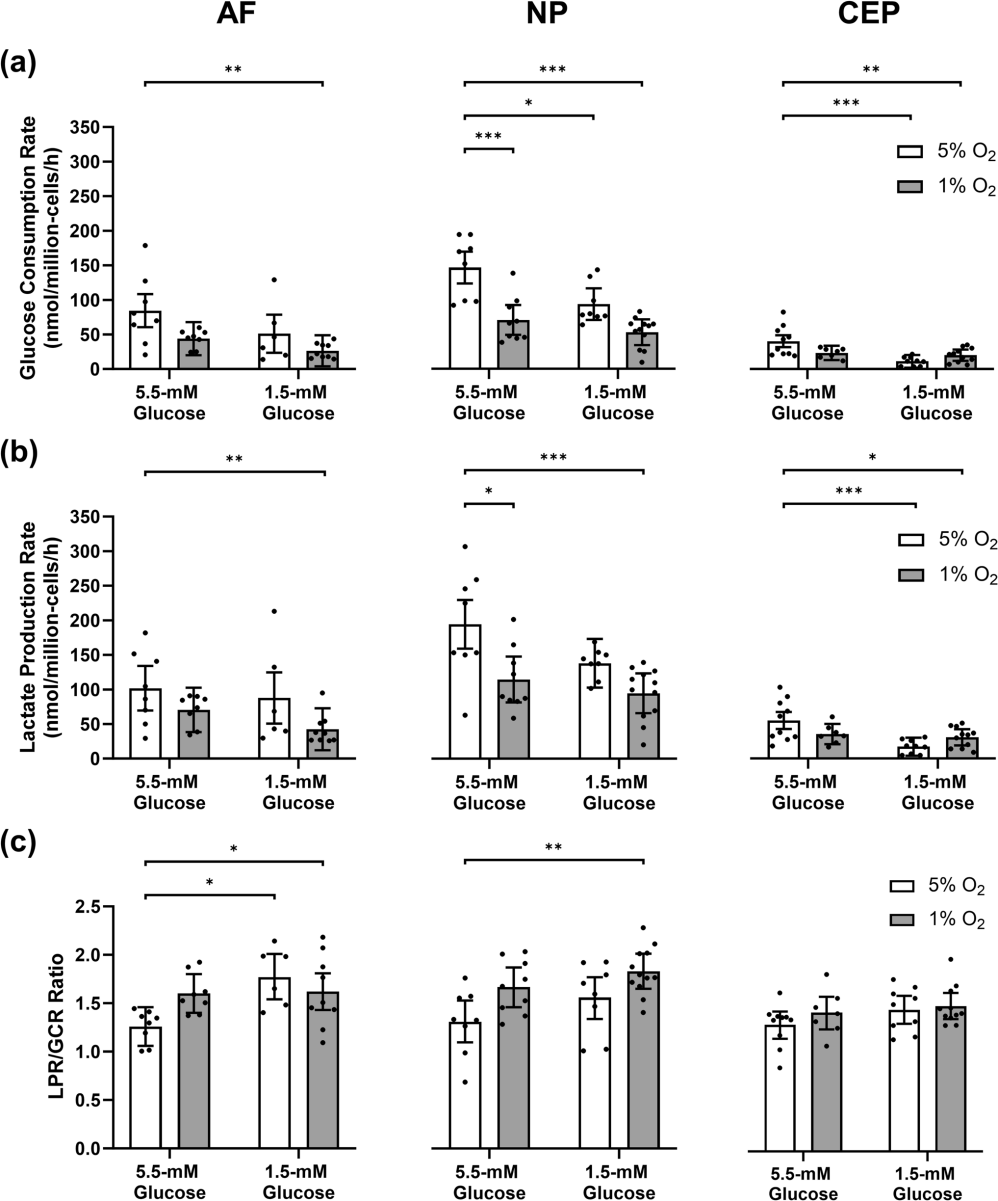
Comparison of the effects of glucose and oxygen levels in IVD culture media on cellular metabolic rates: **a** glucose consumption rate (GCR), **b** lactate production rate (LPR), and **c** cellular energy ratio (LPR:GCR). Each data point represents an average of 2- to 3-well plate replicates measured from a single rat. Asterisks indicate differences at the specified levels of statistical significance: *p < 0.05, **p < 0.01, ***p < 0.001

In the AF, both glucose and oxygen levels in the culture media significantly influenced the GCR response (*p* = 0.0256 and *p* = 0.0098, respectively, Fig. 3a), showing a decline as glucose and oxygen availability decreased. For LPR, the oxygen level had a significant impact (*p* = 0.0254, Fig. 3b), reflecting a pattern similar to that of the GCR. The LPR:GCR ratio was significantly affected by the interaction between glucose and oxygen (*p* = 0.0218), with physiological culture conditions exhibiting a generally lower ratio compared to other conditions. This pattern was predominantly influenced by glucose level (*p* = 0.0167). In the NP, there were no significant interactions between the glucose and oxygen environment. However, glucose and oxygen levels both significantly impacted GCR (*p *= 0.0012 and *p* < 0.0001, respectively; Fig. 3a), LPR (*p* = 0.0182 and *p* = 0.0007, respectively; Fig. 3b), and the LPR:GCR ratio (*p* = 0.0351 and *p* = 0.0040). Overall, NP metabolic activity demonstrated the highest sensitivity to the simultaneous reduction of oxygen and glucose availability. In the CEP, the interaction between glucose and oxygen significantly affected GCR (*p* = 0.0066; Fig. 3a), where a general decline in GCR was associated with reduced glucose availability. A similar significant interaction was noted for LPR (*p* = 0.0135; Fig. 3b), also showing a decreasing pattern with lower glucose levels. The LPR:GCR ratio had no main or interaction effects in the CEP (Fig. 3c).

### Effects of Smoking on the IVD Nutrient Environment

**Fig. 4:**
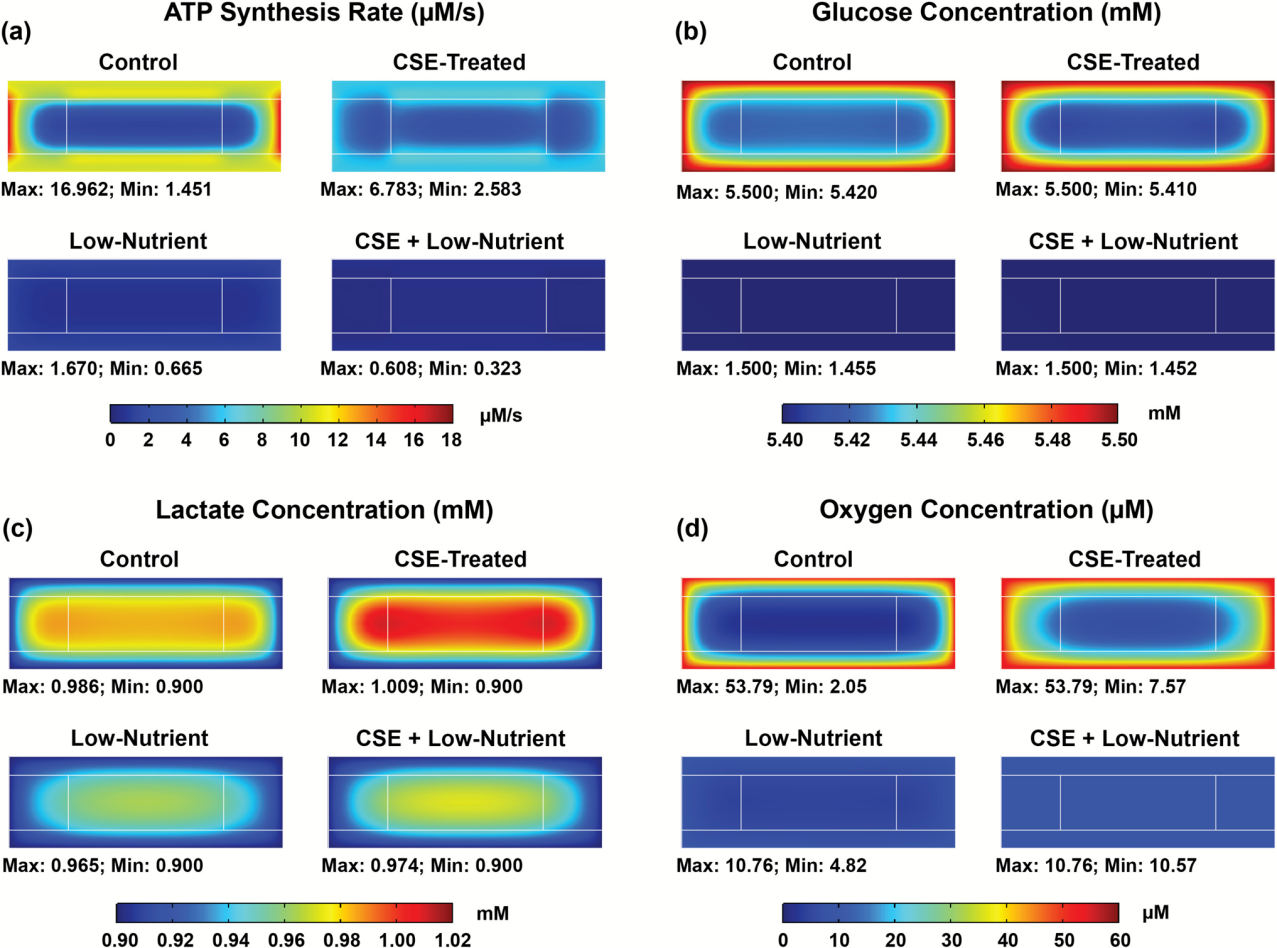
Finite element model of the IVD nutrient environment, visualized on sagittal cross-sections of the IVD. Contours depict local predictions of the **a** ATP synthesis rate, **b** glucose concentration, **c** lactate concentration, and **d** oxygen concentration. Solid white lines indicate regional boundaries between the NP, AF, and CEPs

From the finite element model, qualitatively, the effects of CSE treatment, compared to the physiological case, resulted in shallower concentration gradients for oxygen, glucose, and lactate, particularly in the AF and CEP regions (Fig. 4). In the NP region, however, there were subtle differences in the peak values. The minimum glucose concentration was predicted to be slightly lower with CSE treatment (5.410 mM) compared to the control (5.420 mM, Fig. 4b). Conversely, the maximum lactate concentration was higher with CSE treatment (1.009 mM) than under control conditions (0.986 mM, Fig. 4c). Additionally, oxygen levels were considerably more conserved with CSE treatment, resulting in a higher minimum concentration in the hypoxic NP region (7.57 µM) compared to the control (2.05 µM, Fig. 4d). Although the effects of CSE on nutrient gradients were relatively minor, the reduction in ATP synthesis rate was substantial, with a maximum rate of just 6.783 µM/s for CSE treatment compared to 16.962 µM/s for the control (Fig. 4a).

When comparing nutrient deprivation to physiological conditions, a noticeable decrease in glucose concentration was observed, with a minimum level of 1.455 mM, compared to 5.420 mM in the control group (see Fig. 4b). The lactate production was also reduced, with a maximum concentration of 0.965 mM compared to 0.986 mM (Fig. 4c). Although oxygen levels were depleted overall, the minimum oxygen concentration was slightly higher in the low-nutrient scenario, at 4.82 µM compared to 2.05 µM in the control (Fig. 4d). ATP synthesis was significantly impaired under low-nutrient conditions, with a maximum synthesis rate of 1.670 µM/s compared to 16.962 µM/s in physiological conditions (illustrated in Fig. 4a). This reduction was more severe than the effect observed with CSE treatment, as shown in Fig. 4a. The combined impact of CSE treatment and nutrient deprivation resulted in effects similar to those of CSE treatment under physiological conditions. It is noteworthy that the ATP synthesis rate in this scenario was the lowest overall, with a maximum rate of only 0.608 µM/s.

**Fig. 5:**
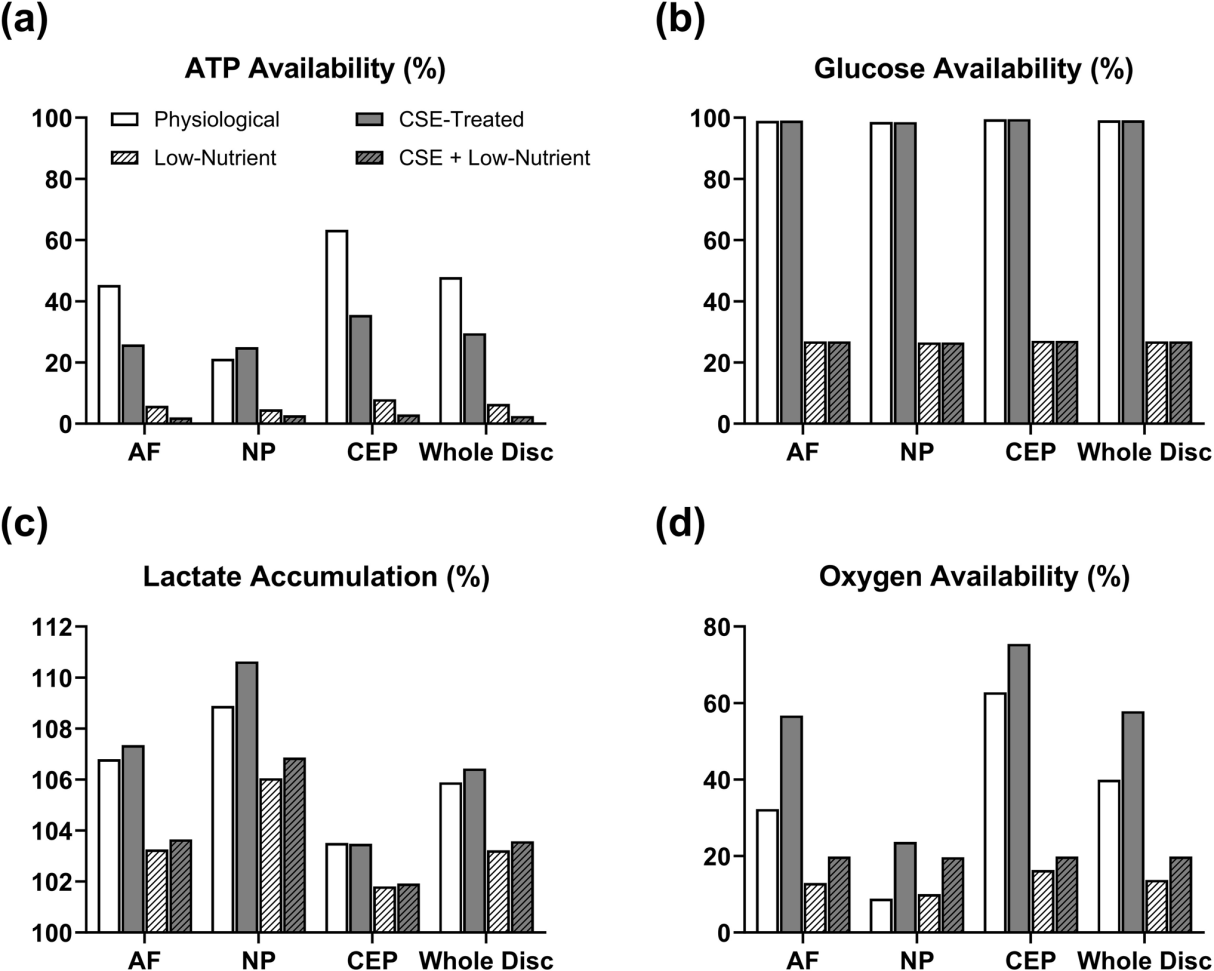
Regional quantitative summary of finite element model outcomes. Bars depict the normalized volumetric integration of **a** ATP synthesis rate, **b** glucose concentration, **c** lactate concentration, and **d** oxygen concentration. Normalization was performed relative to saturation values, calculated under the physiological condition (integrated C_0_ for solute accumulation/availability, or maximum ATP synthesis rate for ATP availability)

A summary of the volumetrically integrated metabolite concentrations and ATP synthesis rates across each IVD region is depicted in Fig. 5. The data were normalized to saturation limits relative to the surrounding environment in the physiological case. Generally, ATP availability decreased in the following order: physiological > CSE-treated > low-nutrient > CSE-treated + low-nutrient (Fig. 5a). In the nucleus pulposus (NP), the ATP synthesis rate showed a slight variation between the CSE-treated and physiological conditions, with slightly higher ATP synthesis observed in the CSE-treated state. Regionally, the largest effects on ATP synthesis due to either nutrient deprivation or CSE treatment occurred in the CEP, followed by the AF, and the NP. In terms of glucose availability, minimal differences were observed between CSE and non-CSE conditions; however, a noticeable decline in glucose levels occurred under low-nutrient conditions, corresponding to the lower boundary value (Fig. 5b). Glucose availability appeared to be consistent across all regions. Increases in both lactate accumulation and oxygen availability were observed with CSE treatment, regardless of the region or conditions of nutrient supply. In contrast, nutrient deprivation had a negative impact on both lactate and oxygen levels (Fig. 5c, d). Regionally, lactate accumulation was highest in the following order: NP > AF > CEP, while oxygen availability followed the opposite trend, with levels highest in the CEP and lowest in the NP. No regional differences in oxygen availability were apparent under combined CSE treatment and low-nutrient conditions.

## Discussion

This study examined the direct (CSE treatment) and indirect (low-nutrient supply) effects of smoking on IVD cellular energy metabolism in SD rat discs. The SD rat animal model has become increasingly relevant for examining the impacts of smoking on IVD degeneration. Recent in vivo studies have used the rat model to examine genetic, cell morphological, and matrix remodeling changes in the IVD [14, 24, 25, 52]. However, metabolic adaptations in this small animal model to smoking remain underexplored. Previous research has quantified glucose consumption rates and lactate production rates in IVDs (Table 1), but these studies have primarily used isolated cells in agarose or alginate gels, focusing on only NP or AF disc regions [33, 34, 33–34]. GCR and LPR averages presently reported from SD disc tissues were consistent with ranges for porcine and bovine discs (Table 1). In a few cases, particularly in the NP, our rates were lower, likely due to the presence of non-notochordal cells (Table 1).

Both direct (CSE treatment) and indirect (low-nutrient) simulated smoking effects diminished metabolic activity across all IVD regions, with significant reductions in the AF and NP. Relative to low-nutrient conditions, CSE-induced decreases in GCR and LPR were less pronounced, suggesting that external nutrient availability primarily influences disc cell energy metabolism (Fig. 2a, b). Both GCR and LPR trended downward (physiological > CSE > low nutrient), but under CSE treatment, only GCR was significantly reduced in both the AF and NP (Fig. 2a, b). This suggests a stronger impact of direct smoking effects on early metabolic processes, such as glucose uptake. Lactate production may be less affected, possibly due to the cells’ use of alternative substrates, such as glutamine, to sustain pyruvate and lactate formation [53]. A study in hepatic cells supports this, showing that both glucose uptake and glycogen storage are reduced with CSE treatment via increased insulin resistance [54]. Similarly, in chondrocytes, reduced glucose uptake due to insulin resistance is implicated in the progression of osteoarthritis [55]. CSE may additionally impair glucose transporters, decreasing cell reliance on extracellular glucose [53, 56, 57]. In terms of energy pathway dependence, the LPR:GCR ratio was found to be higher under both CSE treatment and low-nutrient conditions compared to controls, favoring glycolysis (Fig. 2c). However, this increase was only significant in low-nutrient conditions, likely due to the combined scarcity of glucose and oxygen. In contrast, the milder effect of CSE may result from reduced glucose uptake in an otherwise oxygen-abundant environment.

Regionally, the NP exhibited the highest metabolic activity among the three IVD regions, with significantly elevated GCR and LPR compared to the AF and CEP across all treatments. This aligns with prior literature and may be driven by populations of notochord-like cells in the NP, which are inherently more metabolically active [36, 39]. In contrast, the CEP shows the lowest metabolic activity and greatest resilience to metabolic perturbations, where its chondrocyte-like cells are likely to have lower energy demands. The role of the CEP as a nutritional and mechanical barrier may further support homeostasis amid fluctuating nutrient availability [14, 25, 58]. Notably, the AF, with lower GCR and LPR than the NP, also primarily contains chondrocyte-like cells, suggesting a shared metabolic profile with the CEP [35, 39].

Computational modeling was further employed to understand the spatial interplay between nutrient transport and IVD metabolism in response to smoking (Figs. 4, 5). In the physiological case, gradients of decreasing glucose and oxygen concentrations toward the NP, coupled with increasing lactate concentrations, align with prior IVD nutrient environment models [59–63]. ATP synthesis follows a similar gradient to glucose and oxygen, consistent with other mechanobiological models [50, 64]; however, the gradient is steeper from AF to the NP than from the CEP to the NP. This likely reflects the higher metabolic activity of the AF compared to the CEP, observed in experimental data (Fig. 2a, b). Under CSE-treated conditions, a glucose environment similar to that of the physiological state is predicted; however, ATP production is significantly reduced. This suggests that CSE induces a glycolytic state (higher lactate-to-glucose consumption ratio), feasible only with minimal oxygen consumption (Eq. 6). Consequently, the oxygen environment is preserved, but limited oxygen utilization restricts ATP synthesis to approximately two molecules per glucose molecule. Only one previous modeling study was developed to understand the direct impacts of cigarette smoke. This study modeled steady-state nicotine distributions in the IVD with both light and heavy smoking, and nicotine-mediated changes in cell proliferation and glycosaminoglycan (GAG) biosynthesis [47]. While this model did not examine energy production, the decline in ATP synthesis predicted by the present model would reasonably impact other biosynthetic activities. In a preliminary investigation of GAG biosynthesis using a fluorescent imaging assay, we observed a visual decline in newly synthesized GAG in response to CSE treatment (Online Resource 1, Supp. Fig. 3); however, further work is needed to more thoroughly evaluate this finding [65]. With nutrient deprivation, the dramatic decline in ATP synthesis appeared to derive from the depletion of the glucose environment, leading to lower overall glucose consumption, lactate production, and oxygen consumption. While the effects of CSE on energy synthesis are smaller by comparison, they appear to be additive to those of nutrient deprivation.

This study has several limitations that should be acknowledged. First, the Sprague–Dawley rats that were acquired were not fully skeletally mature at the time of harvest, which limits the generalizability of our findings to other mature animals or to adult human IVD physiology [66–68]. Additionally, the small size of rat discs constrained our ability to measure distinct subregions of the AF, such as the inner versus outer AF, potentially obscuring regional metabolic differences [38]. The exposure duration for the in vitro experiments was limited to 5 h, which may not have been sufficient to elicit sustained metabolic responses, particularly under CSE treatment [39]. Sensitivity of metabolic rates to CSE exposure was modest, suggesting that longer exposure may be required to more accurately model chronic smoking effects. In the in vitro analysis, low-nutrient treatments were not tested in combination with CSE treatment due to concerns over measurement reliability with decreased cell viability. Under low-nutrient conditions alone, very low metabolic rates were observed (< 50 nmol/million cells/h in AF and CEP tissues), and the ratio of lactate production to glucose consumption increased toward limits of what may be considered biologically possible for glycolysis-driven energy production (two lactate molecules produced per glucose molecule consumed). In the computational model, however, regional cell density (and hence viability) was assumed to be constant when calculating metabolic rates across all groups, allowing for a valid comparison of the direct and indirect smoking effects in combination. Modeled ATP synthesis was inferred based on previously validated assumptions of oxidative phosphorylation efficiency, but in vitro characterizations (e.g., using a bioluminescence assay) were not performed [50, 69]. Furthermore, spatial gradients of oxygen and glucose were modeled but could not be experimentally verified within a small, anatomically complex rat IVD, especially while also considering variations in cellular metabolic rates [59, 70]. It should be noted that the finite element model provides a spatial representation of the impacts of experimentally characterized metabolic activity on disc nutrition and energy production; however, it may not accurately reflect the in situ disc nutrient environment. Finally, although decreased glucose consumption and lactate production rates were observed under both direct and indirect smoking conditions, this study does not directly assess downstream biosynthetic or genetic changes in disc cells, which limits mechanistic insight into the effects of smoking on IVD degenerative remodeling.

In summary, the direct and indirect effects of cigarette smoking on regional IVD cellular energy metabolism were compared in vitro. Rates of cellular glucose consumption and lactate production were significantly reduced under both effects, but the reduction was more pronounced with nutrient deprivation (indirect effect) than with CSE treatment (direct effect). This is suggested to be due to the combined scarcity of glucose and oxygen under low-nutrient conditions, as well as the underutilization of oxygen for energy production with CSE treatment. Cells in the NP exhibited the greatest metabolic activity, while those in the CEP were more resilient to environmental disruptions. Aligned with the in vitro findings, computational modeling demonstrated that CSE treatment maintains oxygen levels in the IVD nutrient environment but reduces ATP synthesis, whereas low-nutrient conditions result in a significant decline in ATP synthesis due to the depletion of both glucose and oxygen.

## Supplementary Information

Below is the link to the electronic supplementary material.

Supplementary material 1 (PDF 1839.5 kb)

## Data Availability

The data supporting this study are available within the manuscript and its supplementary files. Raw datasets are available from the corresponding author on reasonable request.
