# Supplementary material for: A Comparison of Cigarette Smoking Effects on Intervertebral Disc Cell Metabolism in a Rat Tissue Model
Source: Ann Biomed Eng. 2026 Jan 20;54(6):1723–37. doi: 10.1007/s10439-025-03958-x (PMC13159066; doi:10.1007/s10439-025-03958-x)
Supplement: Supplementary file 1 — Supplementary material 1 (PDF 1839.5 kb) [file 10439_2025_3958_MOESM1_ESM.pdf]

Supp. Table 1. Metabolic rates measured in high-glucose media (25 mM) under varying oxygen tensions, and in physiological- and low-glucose media at interacting oxygen tensions. Values are reported as mean [95%-CI].

IVD Region	Glucose (mM)	Oxygen (%)	GCR (nmol/million cells/hour)	LPR (nmol/million cells/hour)	Ratio (LPR:GCR)
**AF**	25	5	121 [90.63, 151.37]	140.45 [109.26, 171.64]	1.26 [1.07, 1.45]
**NP**	25	5	256.83 [224.93, 288.73]	257.72 [225.38, 290.06]	1.02 [0.82, 1.22]
**CEP**	25	5	80.49 [50.11, 110.86]	95.77 [64.58, 126.97]	1.22 [1.03, 1.41]
**AF**	25	1	68.33 [44.37, 92.29]	87.72 [63.29, 112.14]	1.31 [1.16, 1.46]
**NP**	25	1	93.92 [68.38, 119.46]	112.43 [86.75, 138.1]	1.21 [1.05, 1.37]
**CEP**	25	1	40.08 [14.54, 65.62]	45.28 [19.61, 70.96]	1.18 [1.01, 1.34]
**AF**	5.5	1	43.84 [20, 67.67]	70.42 [38.31, 102.52]	1.6 [1.4, 1.8]
**NP**	5.5	1	70.74 [49.14, 92.34]	113.81 [80.83, 146.79]	1.66 [1.45, 1.86]
**CEP**	5.5	1	23.27 [12.97, 33.57]	35.09 [20.46, 49.73]	1.49 [1.31, 1.66]
**AF**	1.5	5	51.1 [23.58, 78.62]	87.73 [50.65, 124.8]	1.77 [1.54, 2.01]
**NP**	1.5	5	93.75 [70.84, 116.66]	137.28 [102.3, 172.26]	1.55 [1.33, 1.76]
**CEP**	1.5	5	11.09 [2.01, 20.18]	16.95 [4.04, 29.85]	1.52 [1.37, 1.67]

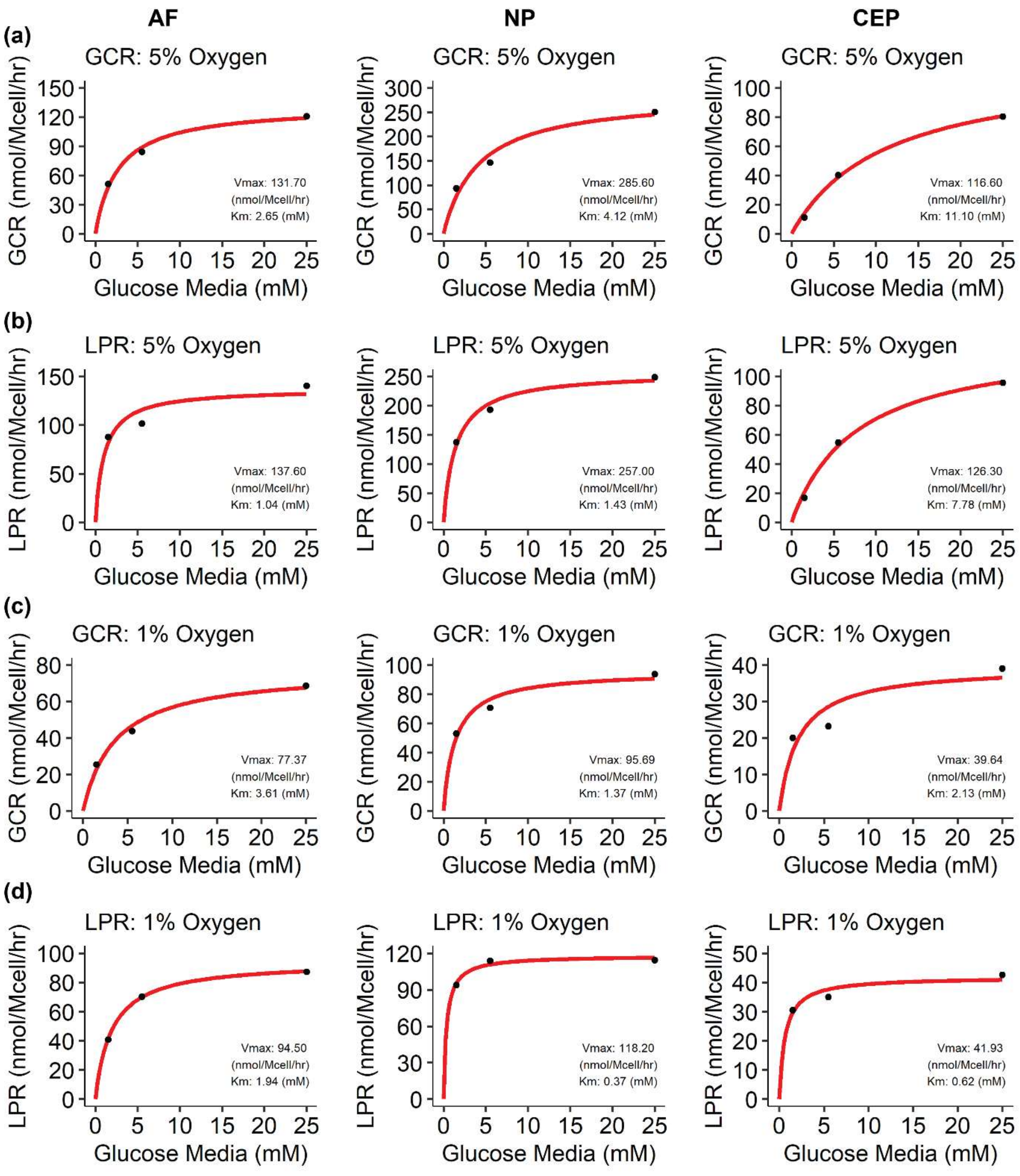

Supp. Figure 1. Michaelis–Menten kinetic models of glucose consumption (a, c) and lactate production (b, d), curve-fit to regional averages of *in vitro* IVD metabolic rates. Models are shown under physiological oxygen tension (a, b) and low oxygen tension (c, d).

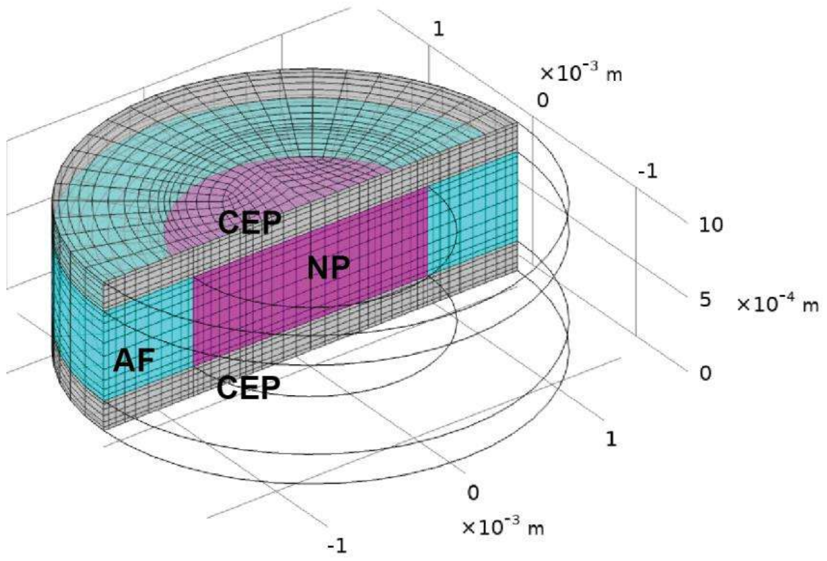

Supp. Figure 2. Geometry used in finite element model simulation of the IVD nutrient environment. Mesh depicts modeled IVD regions, annulus fibrosus (AF), nucleus pulposus (NP), and superior and inferior cartilaginous endplates (CEP), cut at the sagittal plane for visualization. The complete mesh consists of 22,655 nodes, and 21,120 hexahedral volume elements.

Supp. Note 1. Estimation of nutrient solute diffusivity for the finite element model.

Diffusivities of glucose, lactate, and oxygen have not been directly measured in SD rat IVDs. However, the diffusivity of fluorescein (376 Da) in SD rat IVDs has been quantified using a two-dimensional fluorescent recovery after photobleaching (FRAP) [25]. In a separate study on rabbit corneal cartilage, FRAP measurements established a strong linear relationship ($R^2 = 0.99$) between fluorescein diffusivity and molecular weight [2], described by the equation:

$$log(D) = \beta_0 log(MW) + \beta_1$$

Where D is the diffusivity (expressed in $\mu m^2/s$), and MW is the solute molecular weight (expressed in Da), and β_0=-0.40 and β_1=2.44 are regression coefficients derived from publicly available 2D-FRAP data [68]. To estimate nutrient diffusivities in SD rat IVDs, it was assumed that the structural effects of cartilage on solute transport are consistent across different solutes. This assumption leads to the relationship:

$$\frac{D_{IVD\ nutrient}}{D_{cornea\ nutrient}} = \frac{D_{IVD\ fluorescein}}{D_{cornea\ fluorescein}}$$

This approach is supported by a previous study, which showed that, despite variations in cartilaginous tissue composition, solute diffusivity (and its relationship with other solutes) primarily depends on solute molecular weight [69]. A similar simplification was adopted in a prior modeling study [61]. Since the same weight of fluorescein (376 Da) was used in both corneal and IVD studies (including NP, AF, and CEP regions), the ratio $D_{IVD\ fluorescein}/D_{cornea\ fluorescein}$ was known. Using molecular weights of oxygen (16 Da), glucose (180 Da), and lactate (90.1 Da), regional nutrient diffusivities in SD rat IVDs could be estimated.

<u>**Glycosaminoglycan (GAG) biosynthesis.**</u>

A copper-free click chemistry assay was used to evaluate glycosaminoglycan (GAG) synthesis under physiological, CSE-treated, and low-nutrient conditions. Intact whole IVD explants were preconditioned for 48 h in a 12-well plate with high-glucose (25 mM) DMEM supplemented with 1% insulin–transferrin–selenium, 0.9% sodium pyruvate (100 mM), 0.2% ascorbic acid 2-phosphate (500× stock), 0.2% L-proline (500× stock), and 1% antibiotic–antimycotic. Cultures were maintained at 5% CO_2 and 5% O_2, with media refreshed after 24 h. Following preconditioning, explants were treated for 24 h with physiological, CSE, or low-nutrient media as defined in the Methods.

For metabolic labeling, culture media was replaced with fresh media containing 30 µM azide-modified monosaccharide N-azidoacetylgalactosamine (GAL; Invitrogen™ Click-iT metabolic labeling reagents) and incubated for 24 h. GAL is incorporated into intracellular O-linked glycoproteins via the oligosaccharide biosynthesis pathway [62]. Explants were thoroughly washed with PBS and DMEM to remove unincorporated GAL, followed by labeling with 30 µM Alexa Fluor 488 dye (AF488; Invitrogen™ Click-iT detection reagents) for 2.5 h. After labeling, excess dye was removed by repeated washes with PBS and phenol-red-free DMEM, and then explants were incubated overnight [62]. Finally, cell nuclei were stained with SYTO™ Deep Red Fluorescent Nucleic Acid Stain (Invitrogen™ by Thermo Fisher Scientific) for live-cell imaging.

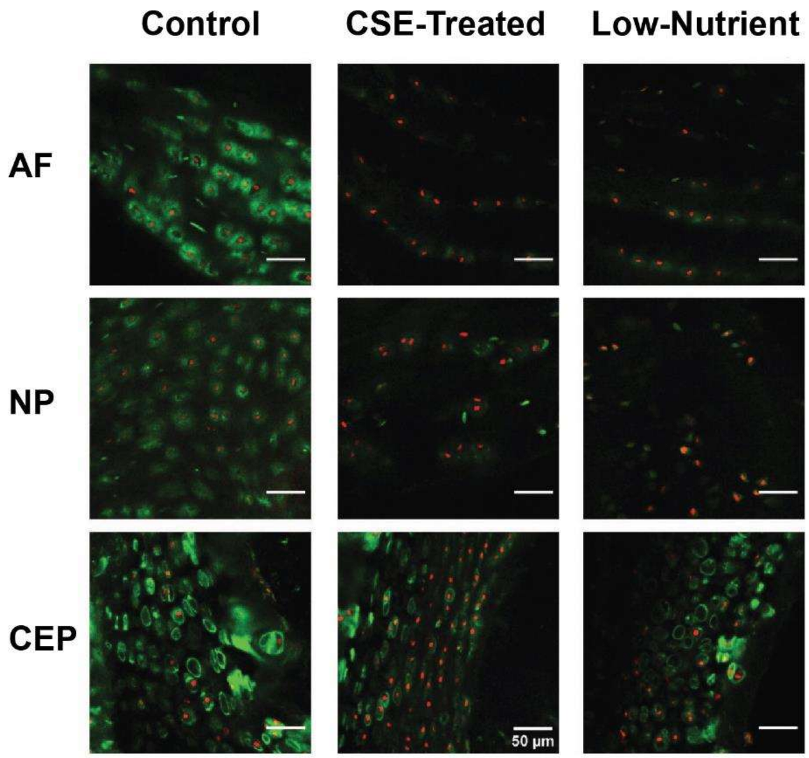

Supp. Figure 3. Fluorescent images of newly synthesized GAG content produced under simulated direct and indirect smoking treatments, captured from representative sections of AF, NP, and CEP tissue. GAG appears as a green halo around red-stained cell nuclei. Scale bars are 50 µm in length. Samples were imaged using a 20X objective on a Leica confocal microscope (DM6000 B, Leica Microsystems, Deerfield, IL) using an Ar-488 laser (excitation wavelength 488 nm). Images were taken approximately 20 µm below the surface of the tissue to avoid capturing cells damaged during preparation.
